# Fluid interaction study: Hydrodynamic robot (FISHR) — Expansion of bioinspired soft robotic fish

**DOI:** 10.1016/j.ohx.2025.e00674

**Published:** 2025-07-16

**Authors:** Montana Ligman, Kioumars A. Rezaie, Ramya Shah, Chris Keeter, Bryson Sutterfield, Mirjam Fürth

**Affiliations:** Texas A&M University, Department of Ocean Engineering, College Station, Tx, 77843, USA

**Keywords:** Biomimetic, Soft robotics, Oscillating propulsion

## Abstract

We introduce an enhanced iteration of OpenFish, a previously developed open-source soft robotic fish. The original model, developed at Delft University of Technology, successfully emulated thunniform swimming through a unique propulsion system utilizing both active and passive tail segments. This design aimed to optimize speed and efficiency while fostering future advancements in soft robotic fish research. To further enhance OpenFish, we undertook a redesign process, making modifications to the fish hull and internal components. These changes aimed to simplify construction, address waterproofing issues, and facilitate the development of an autonomous version of the fish. Our work encompasses an updated description of the construction process, customization options, and detailed insights into hardware implementation, including waterproofing techniques for the soft robotic fish.

## Graphical abstract

**Figure:**
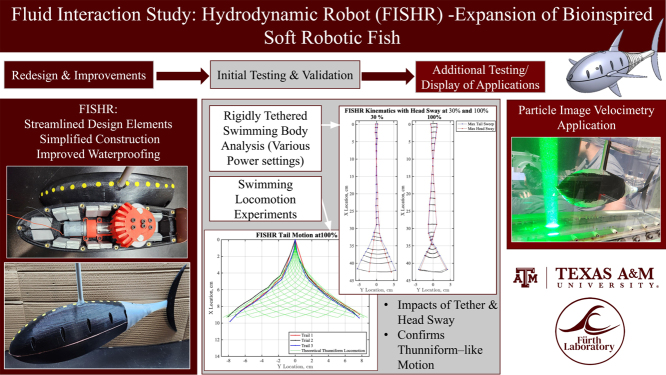

**Table:** 

Hardware name	Fluid Interaction Study: Hydrodynamic Robot

Subject area	•Engineering and material science • Biomimetic robotics

Hardware type	•Ocean engineering • Mechanical engineering and materials science

Closest commercial analog	No commercial analog is available
Open source license	Creative Commons Attribution 4.0 International License (CC BY 4.0).
Cost of hardware	$286.52
Source file repository	https://www.doi.org/10.17632/6sxcdvzn4d.1

	

## 1 Hardware in context

Biomimetics offers a promising approach to sustainability and innovation, as it encourages more resource-efficient and environmentally friendly solutions by emulating the efficiency and elegance of nature’s designs. In recent years, there has been a growing interest in utilizing biomimetic designs such as robotic fish in various fields such as environmental monitoring [1], [2], underwater exploration [3], [4], marine biology and behavior studies of aquatic ecosystems [5], [6], [7], surveillance and security [8], as well as the study of fish swimming mechanics and hydrodynamics [9], [10]. This growing interest has driven the development of aquatic unmanned vehicles and, coupled with biomimetic design, has led to the development of a wide variety of fish-like robots.

Fish demonstrate extraordinary propulsion efficiencies, superior acceleration, and excellent maneuverability [11]. This realization has sparked interest in studying fish morphology and swimming behaviors to integrate these principles into the design of robotic fish, paving the way for the next generation of underwater vehicles [12]. The development of robot fish represents a convergence of diverse research fields, including bionics, mechanics, electronics, automatic control, and material science. In comparison to traditional rigid-form underwater robots like the widely-used Autonomous Underwater Vehicles (AUVs), robot fish offer significant advantages, such as enhanced maneuverability, heightened propulsion efficiency, and reduced noise levels [13]. Although current prototypes of robotic fish exhibit notable achievements, there are still discernible gaps in their performance when compared to real fish.

The rapid evolution of the robotics field has led to the development of numerous robotic fish within biology and biomechanics. However, the absence of a suitable open-source design that can serve as a shared platform for research and development has been a significant limitation. Constructing a robotic fish with a satisfactory performance from scratch is a time-consuming endeavor, often involving extensive fine-tuning of design parameters through literature research and multiple design iterations [14]. This process hampers the efficient dissemination of information across various disciplines, impeding overall progress. Furthermore, the absence of a standardized robotic fish platform has hindered direct comparisons of results due to differences in swimming forms, mechanics, and highly divergent designs.

The OpenFish design, as introduced by [14], offers a fundamental platform that is widely accessible, enabling researchers to customize and create new studies that can be easily replicated and expanded upon by other scientists. The fabrication of the OpenFish design prioritizes accessibility, affordability, and ease of modification, with the ultimate aim of serving as a foundation for future research and development. The works by [14], [15] delve into the optimization of the soft robotic fish with a compliant tail for speed and efficiency. [15] discusses the rationale behind the oscillating propulsion system’s design and the morphology of the robotic fish, drawing on insights from previous literature and modeling. Furthermore, [14] provides a comprehensive description of the open-source OpenFish design, including all the necessary design files, facilitating replication and enabling others to build upon this design.

This paper presents an enhanced model based on the previously developed design, offering further insights into the production and assembly process while addressing specific aspects for improved construction time and waterproofing of the robotic fish. The original model, as introduced by [14], achieved successful emulation of thunniform swimming using a unique propulsion system that incorporates both active and passive tail segments. In our version of the OpenFish, we have streamlined the construction process and resolved waterproofing issues.

Moreover, this updated model undergoes more extensive validation, with an in-depth study of tail kinematics, comparing them to theoretical thunniform locomotion. Additionally, the Open Fish head sway is considered in conjunction with the tail locomotion. In this analysis, a strut was devised to rigidly hold the fish inside a circulating tank, and the wake of the swimming fish was examined at its top speed, both with and without the additional fins.

## 2 Hardware description

**Fig. 1:**
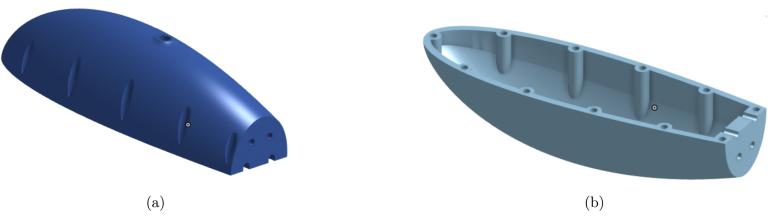
The CAD images of the redesigned Top and Bottom Hull for the FISHR design. (a) Part #1 Top Hull of the FISHR design. The top hull was modified to close the rear end as much as possible to aid in waterproofing. (b) Part #2 Bottom Hull of the FISHR design. The bottom hull was modified by using heat inserts and closing the holes previously used for securing the hulls together.

**Fig. 2:**
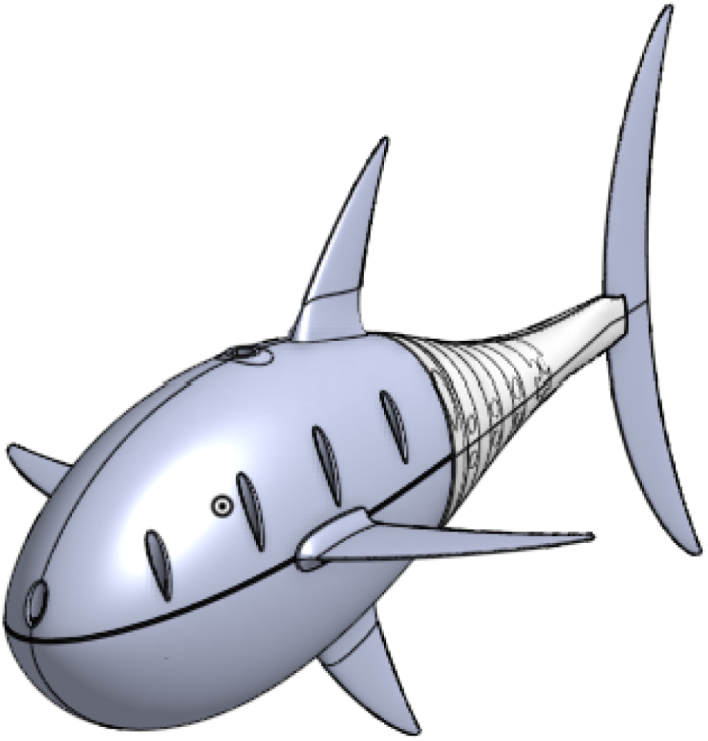
The final CAD assembly of FISHR.

This article introduces an advanced iteration of the open-source soft robotic fish, OpenFish. Originally, OpenFish was crafted to facilitate research on oscillating propulsion, expedite the evolution of autonomous and intelligent soft robotic fish, and support underwater tasks and data collection endeavors [14]. The biomimetic design of OpenFish, mirroring the thunniform swimming pattern, emphasizes speed and efficiency, powered by a single DC motor propelling the oscillation system [14]. Our new design, termed “Fluid Interaction Study: Hydrodynamic Robot” or FISHR, retains the core design tenets and propulsion dynamics of OpenFish but offers enhanced water intrusion resistance and a streamlined assembly process. The redesign achieved a notable reduction in water intrusion vulnerabilities by revamping the fish’s upper and lower hull, as shown in Fig. 1. Specifically, the lower hull has been reimagined to exclude all 11 bolt and nut hole openings, resulting in a seamless structure, bolstering the fish’s waterproof attributes. Concurrently, the top hull’s posterior section has been modified to accommodate a gasket, facilitating a tighter fit with the first tail rib segment. To simplify assembly and disassembly, the bolt holes on the bottom hull have been enlarged and deepened to house threaded heated inserts, suitable for M3 bolts. This adjustment extends to the motor assembly base plate and includes the addition of two threaded inserts at each hull’s end to secure the initial tail rib segment, reinforcing the seal against water entry. Consequently, the first rib tail segment now features a redesigned top with two holes, facilitating its fastening to both hulls using M3 bolts. Further enhancements include the augmentation of the silicone tail’s dimensions, ensuring an improved overlap at the hulls’ rear, fortifying the tail’s integrity, and eliminating potential water ingress. Additionally, the tail mold has been redesigned to feature smoother edges and reinforced areas, minimizing the risk of tears during both the demolding process and operational use. We also increased the silicone tail cover thickness, further enhancing durability. Fig. 2 illustrates the fully assembled FISHR model, incorporating these modifications for improved performance and ease of assembly. This article concludes with a detailed assembly guide for the refined fish model.

While the intent and utilization of FISHR align with those of OpenFish, the new model introduces notable improvements that enhance its performance and capabilities.

The FISHR model advances the foundational work presented in “OpenFish: Biomimetic design of a soft robotic fish for high-speed locomotion” by van den Berg et al. incorporating several vital enhancements that build upon the original design. While the FISHR’s intent and utilization of FISHR align with those of OpenFish, the new model introduces notable improvements that enhance its performance and capabilities.

• **Verification and comparison of research on oscillating propulsion:** The OpenFish and FISHR models play a vital role in validating oscillating propulsion research and evaluating various design elements. Key areas of investigation include the lengths of both the active and passive tails, the rigidity of the compliant tail, the head’s contour, and the fin configuration. The use of 3D-printed components in both models allows for straightforward modifications, making them ideal for testing and comparative studies. Their cost-effectiveness, simplicity in assembly, and ease of control further support the development of test replicas, facilitating rigorous validation and analysis. The FISHR model, however, advances this research by incorporating significant improvements over the OpenFish design. FISHR’s redesigned hull and enhanced waterproofing address previous vulnerabilities, offering greater reliability in experimental setups. The streamlined hull structure and reinforced tail segment of FISHR notably increase its resistance to water ingress, allowing for more precise adjustments to critical design parameters like tail length and rigidity. These advancements enable thorough testing and comparison of different propulsion configurations, thereby enhancing the validation of oscillating propulsion research.
• **Underwater exploration and task performance:** The FISHR model significantly improves underwater exploration and task performance compared to its predecessor. Its optimized hull design and reinforced silicone tail enhance durability and performance in aquatic environments, reducing the risk of water ingress and ensuring reliable operation. These improvements make FISHR particularly suited for exploration, surveillance, and data collection tasks while minimizing disturbance to marine life. The model’s robust construction also supports the integration of additional sensors and data collection tools, making it a valuable asset for studying marine organisms and performing various underwater tasks.

## 3 Design files summary

**Table:** 

Design filename	File type	Open source license	Location of the file
Part #1 Top Hull	STL File	CC.	Available with the article.
Part #2 Bottom Hull	STL File	CC.	Available with the article.
Part #3 Caudal Fin	STL File	CC.	Available with the article.
Part #4 Pectoral Fin	STL File	CC.	Available with the article.
Part #5 Dorsal & Anal Fin	STL File	CC.	Available with the article.
Part #6 Rib Tail Seg. No. 1	STL File	CC.	Available with the article.
Part #7 Rib Tail Seg. No. 2	STL File	CC.	Available with the article.
Part #8 Rib Tail Seg. No. 3	STL File	CC.	Available with the article.
Part #9 Rib Tail Seg. No. 4	STL File	CC.	Available with the article.
Part #10 Silicone Tail	SLDPRT File	CC.	Available with the article.
Part #11 Left/Right Outer Tail Mold	STL File	CC.	Available with the article.
Part #12 Inner Tail Mold	STL File	CC.	Available with the article.
Part #13 Insert for Inner Tail Mold	STL File	CC.	Available with the article.
Part #14 Hull Gasket Template	STL File	CC.	Available with the article.
Part #15 Motor Assembly Base Plate	STL File	CC.	Available with the article.
Part #16 Gearbox Plate Mount	STL File	CC.	Available with the article.
Part #17 Gearbox Assembly Mount	STL File	CC.	Available with the article.
Part #18 Tail Spine	STL File	CC.	Available with the article.
Part #19 Gearbox Assembly Mounting Plate	STL File	CC.	Available with the article.
Part #20 Pushrod Connector Plate	STL File	CC.	Available with the article.
Part #21 Fish Mount Front Plate	STL File	CC.	Available with the article.
Part #22 Fish Mount Back Plate	STL File	CC.	Available with the article.
Part #23 Fish Mount Side Plate	STL File	CC.	Available with the article.

Part #1 Top Hull component which will be secured to the bottom hull.
Part #2 Bottom Hull component which will be secured to the top hull.
Part #3 Caudal Fin component which produces the force required to propel FISHR.
Part #4 Pectoral Fin component consists of the left and right fin.
Part #5 Dorsal and Anal Fin components which are attached to the top and bottom hull and to the tail spine.
Part #6 Rib Tail Segment No. 1 (largest segment) component which is secured to the top and bottom hull.
Part #7 Rib Tail Segment No. 2 component which is secured to the tail spine.
Part #8 Rib Tail Segment No. 3 which is secured to the tail spine.
Part #9 Rib Tail Segment No. 4 (smallest segment) component which is secured to the tail spine.
Part #10 Silicone tail formed from parts #11, 12, and 13.
Part #11 Left and Right outer tail mold used to mold the silicone tail.
Part #12 Inner tail mold.
Part #13 Insert for inner tail mold part #12.
Part #14 Hull gasket template to trace part #41.
Part #15 Motor assembly base plate component which houses the motor, coupler, and L-drive.
Part #16 Gearbox plate mount component.
Part #17 Gearbox assembly mount component.
Part #18 Tail spine, this consists of two parts.
Part #19 Gearbox assembly mounting plate component.
Part #20 Pushrod connector plate, where the pushrod connectors part #31, are attached.
Part #21 Fish mount front plate.
Part #22 Fish mount side plate.
Part #23 Fish mount side plates.

## 4 Bill of materials summary

**Table:** 

Designator	Component	Number	Cost per unit- U.S. Dollar	Total cost- currency	Source of materials	Material type
Part #1	Top Hull	1	$19.99/kg	$4.09 (205 g)	TAMU-Dept of Mech Eng${}^{*}$	PLA

Part #2	Bottom Hull	1	$19.99/kg	$2.15 (108 g)	TAMU	PLA

Part #3	Large Caudal Fin	1	$19.99/kg	$0.29 (14.5 g)	TAMU-Dept of Mech Eng${}^{*}$	PLA

Part #4	Left and Right Pectoral Fin	2	$19.99/kg	$0.40 (20 g)	TAMU-Dept of Mech Eng${}^{*}$	PLA

Part #5	Dorsal and Anal Fin	2	$19.99/kg	$0.34 (17.2 g)	TAMU-Dept of Mech Eng${}^{*}$	PLA

Part #6	Rib Tail Segment No. 1 (Largest)	1	$19.99/kg	$0.33 (16.4 g)	TAMU-Dept of Mech Eng${}^{*}$	PLA

Part #7	Rib Tail Segment No. 2 (2nd Largest)	1	$19.99/kg	$0.15 (7.3 g)	TAMU-Dept of Mech Eng${}^{*}$	PLA

Part #8	Rib Tail Segment No. 3 (3rd Largest)	1	$19.99/kg	$0.11 (5.4 g)	TAMU-Dept of Mech Eng${}^{*}$	PLA

Part #9	Rib Tail Segment No. 4 (Smallest and Last)	1	$19.99/kg	$0.12 (5.9 g)	TAMU-Dept of Mech Eng${}^{*}$	PLA

Part #10	2-Part Silicone Tail Cover	1	$19.39/580 g	$1.87 (56 g)	Amazon	Silicone-Shore 15

Part #11	Left/Right Outer Tail mold to cast Part #10	2	$19.99/kg	$6.02 (301.3 g)	TAMU-Dept of Mech Eng${}^{*}$	PLA

Part #12	Inner Tail Mold to cast Part #10	1	$19.99/kg	$3.35 (167.8 g)	TAMU-Dept of Mech Eng${}^{*}$	PLA

Part #13	Insert for the inner tail mold to cast Part #10	1	$19.99/kg	$0.04 (1.8 g)	TAMU-Dept of Mech Eng${}^{*}$	PLA 1 mm thickness

Part #14	Hull Gasket Template	1	$19.99/kg	$0.10 (4.9 g)	TAMU-Dept of Mech Eng${}^{*}$	PLA 1 mm thickness

Part #15	Motor Assembly Base Plate	1	$19.99/kg	$0.30 (14.9 g)	TAMU-Dept of Mech Eng${}^{*}$	PLA

Part #16	Gearbox Plate Mount	1	$19.99/kg	$0.02 (1.2 g)	TAMU-Dept of Mech Eng${}^{*}$	PLA

Part #17	Gearbox Assembly Mount	1	$19.99/kg	$0.36 (18.1 g)	TAMU-Dept of Mech Eng${}^{*}$	PLA

Part #18	Tail Spine	1	$19.99/kg	$0.8 (3.9 g)	TAMU-Dept of Mech Eng${}^{*}$	PETG 1 mm thickness

Part #19	Gearbox Assembly Mounting Plate	2	$19.99/kg	$0.19 (6.7 g)	TAMU-Dept of Mech Eng${}^{*}$	POM 2 mm thickness

Part #20	Pushrod Connector Plate	2	$19.99/kg	$0.04 (2.1 g )	TAMU-Dept of Mech Eng${}^{*}$	POM 2 mm thickness

Part #21	Fish Mount Front Plate	1	$19.99/kg	$0.35 (17.5 g)	TAMU-Dept of Mech Eng${}^{*}$	POM 2 mm thickness

Part #22	Fish Mount Back Plate	1	$19.99/kg	$0.38 (19 g)	TAMU-Dept of Mech Eng${}^{*}$	POM 2 mm thickness

Part #23	Fish Mount Side Plates	2	$19.99/kg	$0.84 (42 g)	TAMU-Dept of Mech Eng${}^{*}$	POM 2 mm thickness

Part #24a	12 V DC Motor 9.7:1	1	$28.95	$28.95	Pololu	Other

Part #24b	12 V DC Motor w/ Encoder	1	$14.88	$14.88	Amazon	Other

Part #25	Motorshaft Coupler 4 × 4 mm	1	$7.99	$7.99	Amazon	Other

Part #26	M0.5 20T Spur Gear	1	$4.51	$4.51	McMaster	Acetal Plastic

Part #27	M0.5 50T Spur Gear	2	$5.19/each	$10.38	McMaster	Acetyl Plastic

Part #28	L-Gearbox 1:1 gear ratio	1	$62.70	$62.70	RSDelivers	Other

Part #29	M5 Flat Washer	2	$6.49/50 pcs	$0.26	Amazon	Nylon

Part #30	Assorted M3 Spacers	6	$9.99/100 pcs	$0.60	Amazon	Nylon

Part #31	D1.3 mm Pushrod Connector	2	$8.97/25 pcs	$0.72	Amazon	Stainless Steel

Part #32	M5 × 20 mm Binding Screws	2	$6.99/5 pcs	$2.80	Amazon	Stainless Steel

Part #33	M3 Assorted Bolts, Nuts, Washers	40	$28.68/1500 pcs	$0.76	Amazon	Alloy Steel

Part #34	M3 Threaded Heated Inserts	19	$9.99/120 pcs	$1.58	Amazon	Brass

Part #35	Eagle Claw 12${}^{\prime \prime }$ 30 lb Braided Fishing Leader	1	$2.32	$2.32	Walmart	Braided Wire

Part #36	Adhesive Wheel Weights 1/2 oz	60	$23.99/90 pcs	$15.99	Amazon	Steel

Part #37	12 V Battery Pack	1	$39.99	$39.99	Amazon	Other

Part #38	DC Motor Controller	1	$19.96	$19.96	Amazon	Other

Part #39	30-Gauge Red/Black Wire	1	$9.98/50 ft	$9.98	Amazon	Silicone Copper

Part #40	Clamp Set	20	$13.96/20 pcs	$13.96	Amazon	Other

Part #41	Silicone Rubber Sheet	1	$12.99/12 × 12 in	$12.99	Amazon	Silicone Rubber

Part #42	Gorilla Glue “Bonding Waterproof, Quick Dry Adhesive”	1	$6.58	$6.58	Lowes	Contact Adhesive

Part #43	LOCTITE Gel Super Glue	1	$5.98	$5.98	Lowes	Contact Adhesive

In order to further assist in obtaining all of the non-3D printed parts listed above, Appendix, included at the bottom of this article, has all of the respective parts’ URLs. It should be noted that the L-gearbox purchased from RS has since been discontinued. A link to the model which replaced the gearbox from RS has been provided. (Updated L-Gearbox from RS: Huco 332.31.2 Huco L Gearbox, 1:1 Gear Ratio, 0.68 Nm Maximum Torque priced at $52.60).

The 3D-printed parts listed in the Bill of materials were fabricated using standard PLA filament provided by the Texas A&M University (TAMU) Department of Mechanical Engineering. The specific brand or type of PLA filament used during the build is not critical to the structure’s integrity or function. However, proper print settings and post-print waterproofing techniques, such as applying coatings or sealants, are essential to ensure the parts’ durability and performance in aquatic environments.

${}^{*}$ The prints were produced in a workshop studio managed by the TAMU Department of Mechanical Engineering, accessible to all faculty and students for educational and research purposes. The department facilitated both the material and labor costs for printing and the PLA filament was sourced directly from their available supplies at the time of production.

## 5 Build instructions

The construction of the FISHR is separated into five phases: motor and gearbox mount assembly, gearbox assembly, hull assembly, tail assembly, and final assembly:

(Note: use of drill bits may be required to bore out some of the printed parts in order to ease assembly.)

**Phase One: Motor and Gearbox Mount Assembly**

**Fig. 3:**
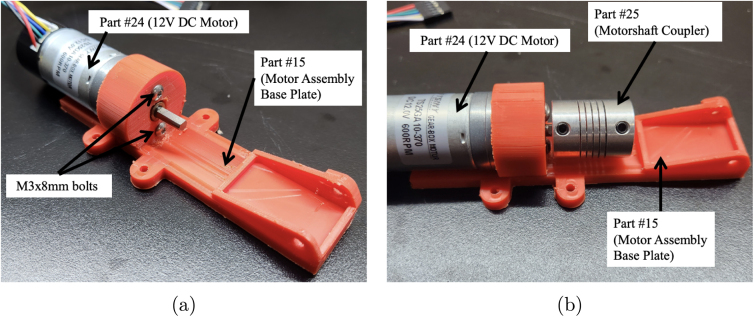
Assembly process for mounting the motor, coupler. The steps (one and two) include securing the motor to the base plate and attaching the motorshaft coupler to the motor. (a) Example photo from step one, which includes securing the motor (part #24a or #24b) to the motor assembly base plate (part #15) using two M3 × 8 mm. (b) Example photo from step two, which includes attaching the motorshaft coupler (part #25) to the motor shaft.

**Fig. 4:**
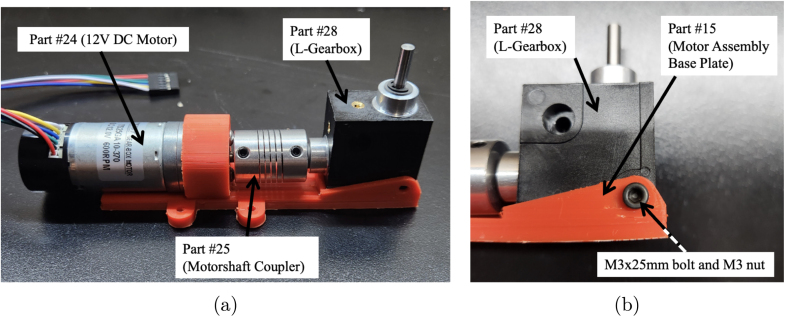
Assembly process for steps three and four, which involves mounting the L-Gearbox onto the motor assembly base plate. The steps include aligning the L-Gearbox with the coupler and fastening the gearbox to the base plate. (a) Example photo from step three, which includes aligning the L-Gearbox (part #28) with the coupler (part #25) and tightening the set screws to secure both shafts. (b) Example photo from step four, which includes fastening the L-Gearbox (part #28) to the base plate (part #15) using an M3 × 25 mm bolt and M3 nut.

**Fig. 5:**
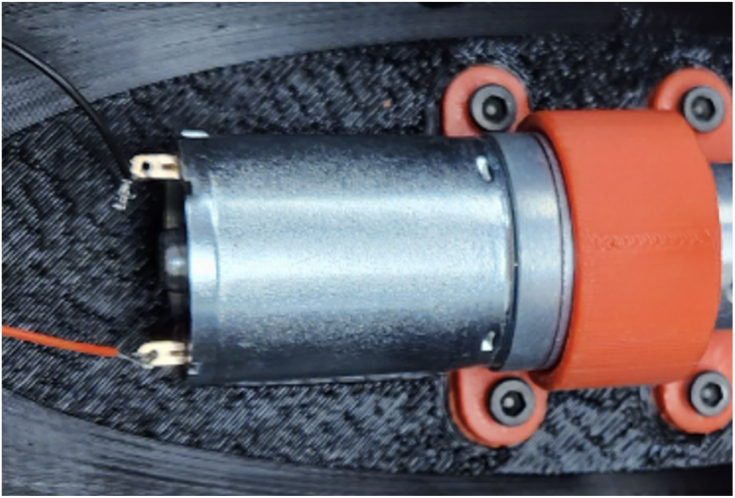
Reference Image to show part #39 (30-Gauge Red/Black Wire) and solder to the respective positive and negative terminals on part #24a (12 V DC Motor 9.7:1). (For interpretation of the references to color in this figure legend, the reader is referred to the web version of this article.)

• The following parts are required for phase one assembly: #15, #17, #24, #25, #26, #28, #30, #33, #39.

1. Take part #24a, or #24b if a motor with encoder is needed, and bolt it to part #15 (Motor Assembly Base Plate). Use two M3 × 8 mm bolts from part #33. Ensure that the motor is securely fastened to the base plate to prevent any movement during operation. (Refer to Fig. 3(a))
2. Place part #25 (Motorshaft Coupler, 4 × 4 mm) over the shaft of the motor #24a or #24b. The coupler should slide easily onto the shaft, but ensure a snug fit to avoid slipping. (Refer to Fig. 3(b))
3. Place part #28 (L-Gearbox), and align it with part #25 (Motorshaft Coupler). Use a hex key to tighten the four set screws on part #25 (Motorshaft Coupler), securing it to the shafts of parts #24 (12 V DC Motor) and #28 (L-Gearbox). This step ensures that the motor’s torque is transferred effectively to the gearbox. (Refer to Fig. 4(a))
4. Secure part #28 (L-Gearbox), to part #15 (Motor Assembly Base Plate), using one M3 × 25 mm bolt and one M3 nut from part #33. Make sure the gearbox is tightly mounted to prevent any misalignment or loosening during operation. (Refer to Fig. 4(b))
5. Take part #26 (M0.5 20T Spur Gear) and place it onto part #28 (L-Gearbox) drive shaft.
6. Using a soldering iron, take part #39 (30-Gauge Red/Black Wire) and solder to the respective positive and negative terminals on part #24a. Fig. 5 shows what the motor with the soldered wires should look like. **Note**: The length of the wire will be dependent on use.

The assembly of the motor and gearbox mount is now complete

**Phase Two: Gearbox Assembly**

**Fig. 6:**
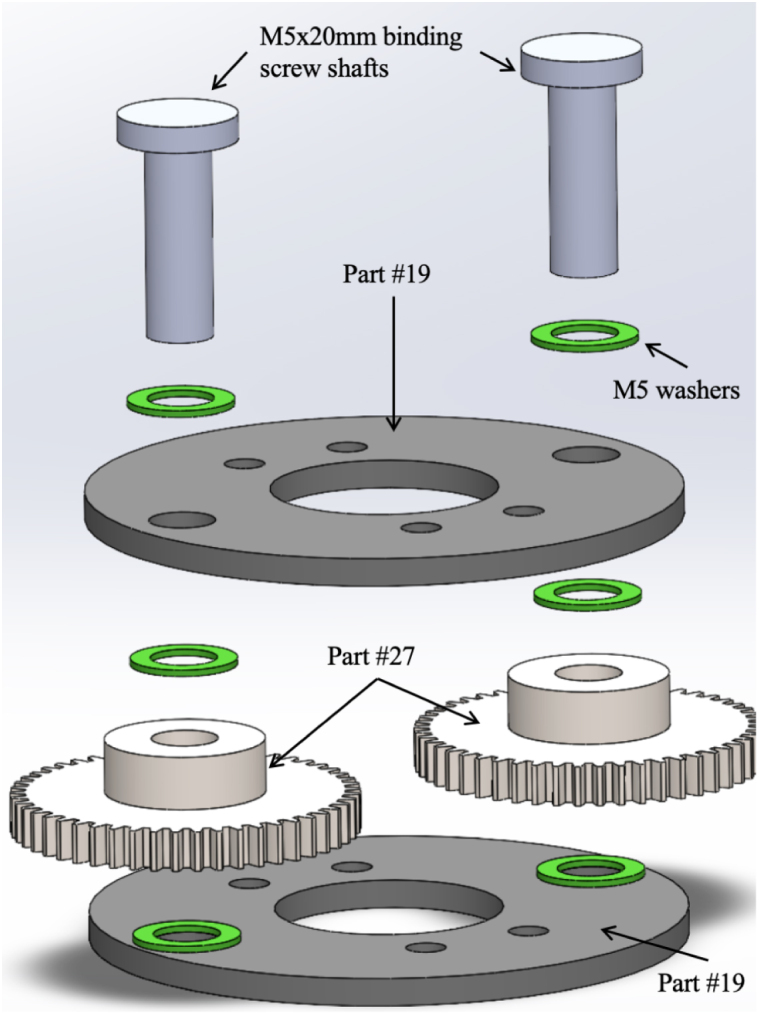
Exploded view of assembly for the gearbox, including steps one through five. Steps include the following parts: two #19 (Gearbox Assembly Mounting Plates), two M5 × 20 mm binding screw shafts from part #32, six #29 (M5 washers), and two #27 (M0.5 50T Spur Gears).

**Fig. 7:**
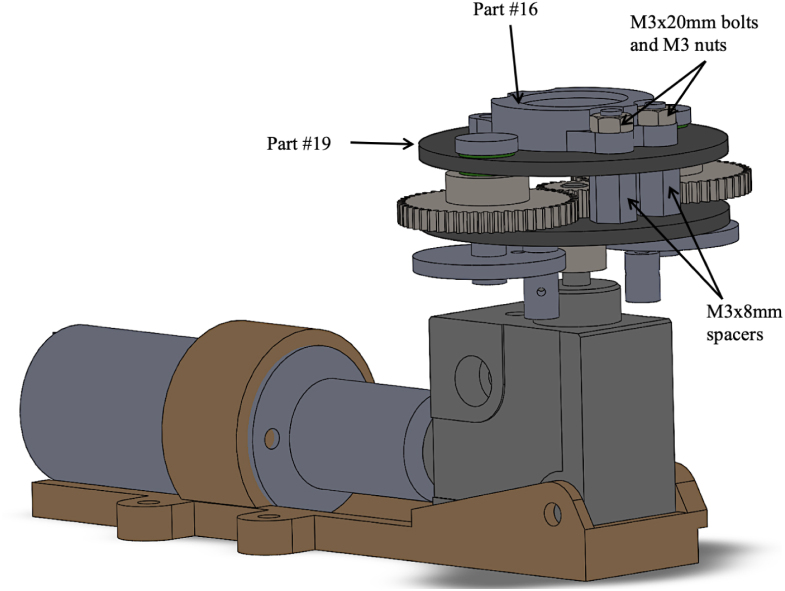
Detailed view of assembly for the gearbox, including step six. Steps include the following parts: one #16 (Gearbox Plate Mount), two #19 (Gearbox Assembly Mounting Plates), two M3 × 20 mm bolts, two M3 nuts, and two M3 × 8 mm spacers. This view shows the partially assembled gear over part #26 (M0.5 20T Spur Gear).

**Fig. 8:**
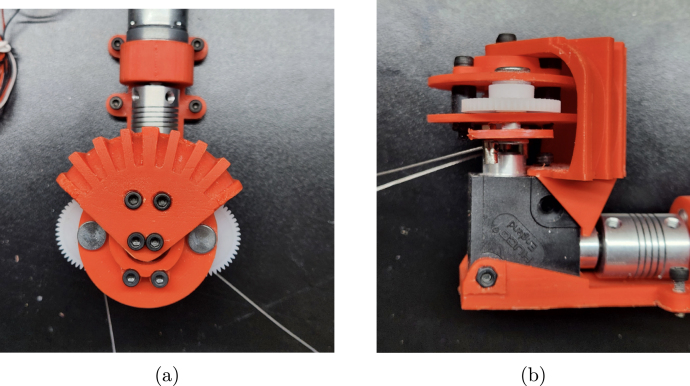
Example photos of the gearbox assembly from step eight, including the placement and securing the part # 17 (Gearbox Assembly Mount). (a) Top view of the part # 17 (Gearbox Assembly Mount) and the required placement of the two M3 × 25 mm bolts and nuts placed in step eight. (b) Side view of the part # 17 (Gearbox Assembly Mount), the gear box assembly, and the required placement of the two M3 × 25 mm bolts and nuts placed in step eight.

**Fig. 9:**
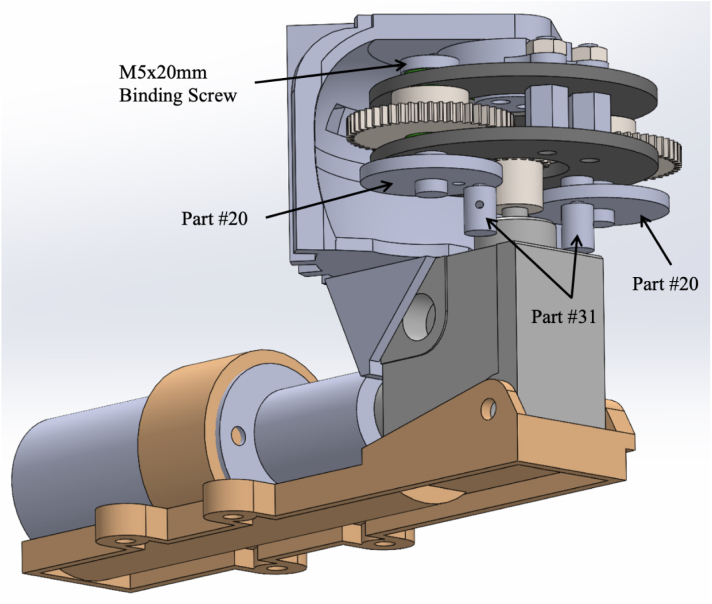
Detailed view of assembly for the gearbox, including steps nine and ten. Steps include the following parts: two M5 × 20 mm Binding Screws, two #20 (Pushrod Connector Plates), and two #31 (D1.3 mm Pushrod Connectors).

• The following parts are required for phase two assembly: #16, 2x #19, 2x #20, 2x #27, 2x #29, #30, 2x #31, 2x #32, #33, #35

1. Take one part #19 (Gearbox Assembly Mounting Plate) and place two M5 × 20 mm binding screw shafts from part #32, through each of the larger diameter holes on the Gearbox Assembly Plate. Make sure to place two #29 (M5 washers) over the shafts before inserting the binding screws into the plate. Fig. 6 Shows the exploded view of this step. **Tip**: Use a 7/32in drill bit to slightly enlarge the diameter of the binding screw holes on part #19 (Gearbox Assembly Mounting Plate) to allow for easier insertion of the binding screw shafts. **Note**: The Gearbox Assembly Plate with the binding screw shafts is now considered the top plate.
2. After placing the two binding screw shafts through the gearbox assembly plate; place two part #29 (M5 washers) over the shafts. See Fig. 6 for a detailed view.
3. Slide part #27 (M0.5 50T Spur Gear) over each of the binding screw shafts. See Fig. 6 for a detailed view.
4. Connect the binding screw and spur gear by drilling a 1/16” hole through the center of each. Place a small screw through the center. This prevents the spur gear from freely rotating around the binding screw.
5. Now place the other part #19 (Gearbox Assembly Plate) over the remainder of the binding screw shaft. See Fig. 6 for a detailed view. **Note**: Between both part #19’s (Gearbox Assembly Mounting Plates) there should be two spur gears and a total of four washers.
6. Place part #16 (Gearbox Plate Mount) on the top of part #19 (Gearbox Assembly Mounting Plate). The non-cut out portion of the Gearbox Plate Mount should be facing toward the tail end of the fish. Place two M3 × 20 mm bolts from part #33 through the top of part #16 (Gearbox Plate Mount) and #19 (Gearbox Assembly Mounting Plate), with two M3 × 8 mm spacers in between the top and bottom Gearbox Assembly Plates. Secure the two bolts using M3 nuts. Now slide this partially assembled gear over part #26 (M0.5 20T Spur Gear), which is sitting on the drive shaft of part #28 (L-Gearbox). See Fig. 7 for a detailed view. **Tip**: Use a 1/8 in drill bit to enlarge the diameter of the holes the two M3/20 mm bolts are going through for ease of assembly.
7. Secure part #17 (Gearbox Assembly Mount), to part #28 (L-Gearbox), using two M3 × 4 mm bolts from part #33.
8. Using the two center holes on part # 17 (Gearbox Assembly Mount), insert two M3 × 25 mm bolts with two M3 × 3 mm spacers between part #17 (Gearbox Assembly Mount) and part #16 (Gearbox Plate Mount). Then, place two M3 × 8 mm spacers between part #19 (Gearbox Assembly Mounting Plate) followed by two M3 × 25 mm bolts all the way through to the bottom plate of part #19 (Gearbox Assembly Mounting Plate). Secure the bolts with M3 nuts. Fig. 8 shows the assembled gearbox, including part # 17 (Gearbox Assembly Mount), and where the corresponding bolts go in the assembly. **Tip**: Use a 1/8 in drill bit to slightly enlarge the diameter of the holes the two M3 × 25 mm bolts are going through.
9. Now take the screw end of part #32 (M5 × 20 mm Binding Screws) and place it through the center hole of both part #20 (Pushrod Connector Plates). Screw the matching female screw post (the “nut”) onto the threaded end to secure the materials together. See Fig. 9 for a detailed view of the assembly. **Tip**: Use a 9/64 in drill bit to slightly enlarge the diameter of the center hole for part #20 in order to allow for the smoother insertion of the binding screw.
10. On both part #20 (Pushrod Connector Plates), insert part #31 (D1.3 mm Pushrod Connector) through the outer hole of the pushrod connector plates. See Fig. 9 for a detailed view of the assembly. **Note**: The pushrod connector should be able to freely spin, so slightly enlarging the outer hole with a drill bit is recommended. Also, placing a dab of part #43 (LOCTITE super glue) on the top nut of the pushrod connector will assist in keeping it from unscrewing once the fish is operational.
11. Take part #35 (Eagle Claw 12” 30lb Braided Fishing Leader). Remove all the attachments and cut them in half so you are left with approximately two 6-inch pieces of steel braided wire.
12. Place the two pieces of part #35 through each of part #31 pushrod connectors, and tighten the wire snug so it cannot be pulled out. **Tip**: leave only a small portion sticking out one end of the pushrod connector. Use part #43 (LOCTITE super glue) to assist in keeping the wire in the pushrod connector.
13. Once the pushrod connector and wire are attached to part #20 (Pushrod Connector Plate), connect the screw end of part #32 (M5 × 20 mm Binding Screws) to the shaft of the binding screw. **Tip**: Place a small amount of part #43 (LOCTITE super glue) on the threads of the binding screw, so it will not back out once the fish is operational.

**Fig. 10:**
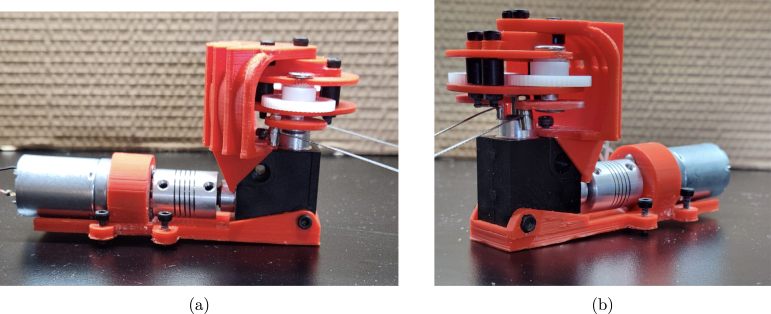
Assembled gearbox attached to the motor and gearbox mount. (a) Profile the fully assembled gearbox and motor ready for installation. (b) Additional profile of the fully assembled gearbox and motor ready for installation.

The assembly of the gearbox is now complete and attached to the motor and gearbox mount. The fully assembled gearbox is shown in Fig. 10.

**Phase Three: Hull Assembly**

**Fig. 11:**
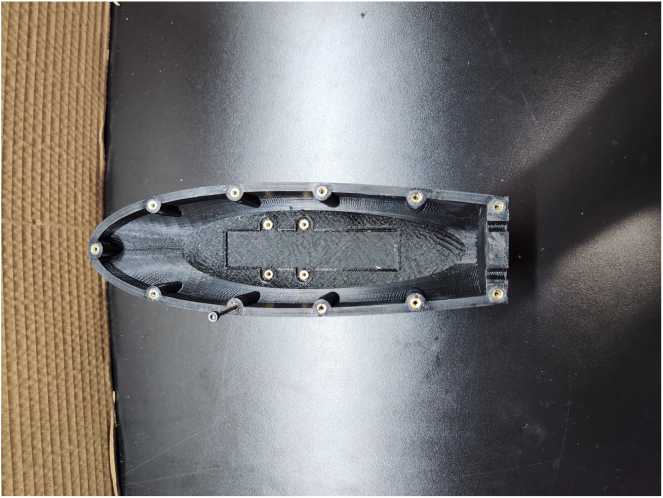
Example image of the M3 Threaded Heated Inserts on Part #2 (Bottom Hull).

**Fig. 12:**
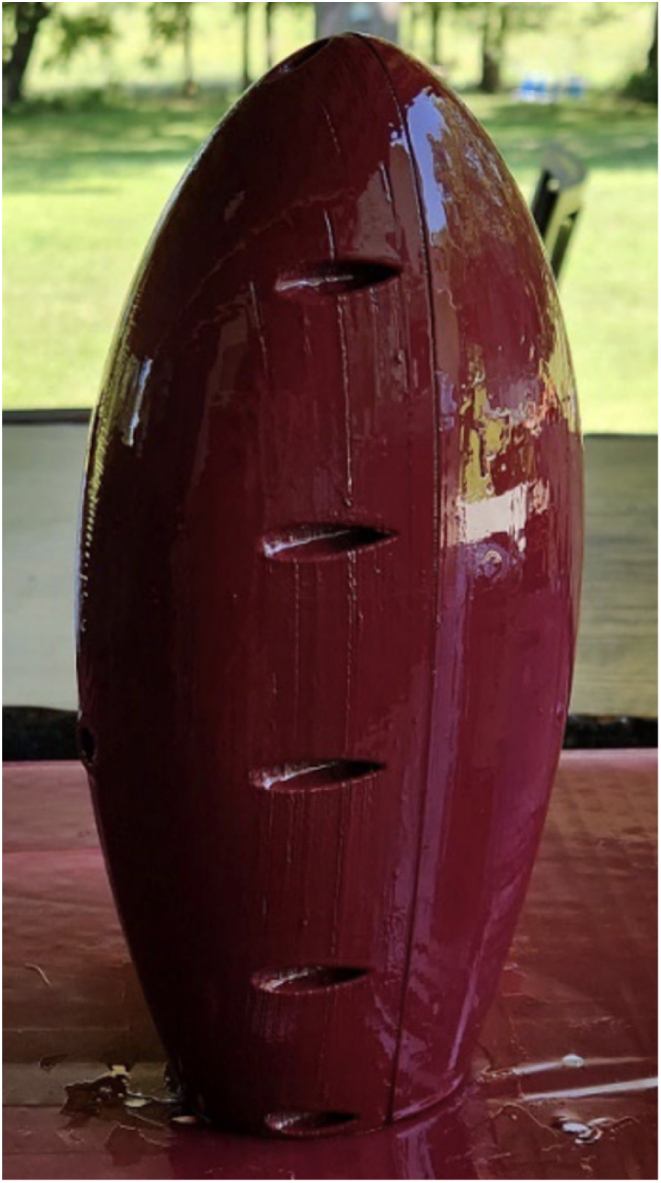
Example of the drying process of the coats of polyurethane to parts #1 and #2.

**Fig. 13:**
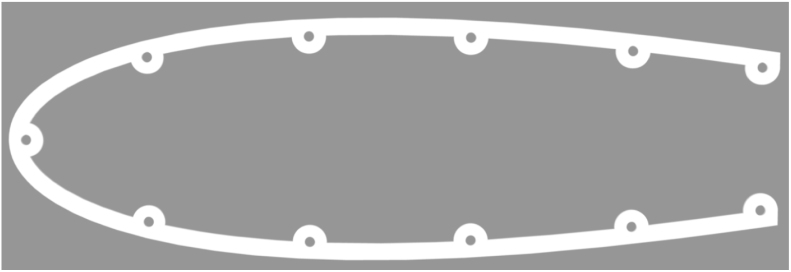
Gasket template.

• The following parts are required for phase three assembly: #1, #2, #14, #34, #41 .
• Remove all residual supports used during 3-D printing.
• The application and utilization of the FISHR model can vary; therefore, the type of strut or flexible conduit that is connected to the top hole of part #1 will be dependent on the intended use of the FISHR.

1. Take part #2 (Bottom Hull); while using a soldering iron, heat up and place part #34 (M3 Threaded Heated Inserts) in 11 of the larger diameter holes around the edge of the hull. Place four heated inserts in the base of the hull where the motor and gearbox will be mounted. At the aft end of the hull, place two heated inserts where part #6 (Tail Segment Rib). Fig. 11 shows what he bottom hull should look like after this step.
2. In a similar process to step two, take part #1 (Top Hull) and place two part #34 (M3 Threaded Heated Inserts) in the larger diameter holes on the aft end of the hull.
3. Apply at least four coats of polyurethane to parts #1 (Top Hull)and #2 (Bottom Hull) using a foam brush in order to get even and consistent layers. Individual coats take approximately 2-4 h to dry before applying another. This will assist in preventing water intrusion into the hull. Fig. 12 shows the applied polyurethane coats to the hulls during the drying process.
4. Use part #14 (Gasket Template) to trace the outline of the gasket on part #41 (Silicon Rubber Sheet). Cut out the gasket and set aside once both hulls are tightened. Fig. 13 shows the template used to trace the gasket shape.

**Phase Four: Tail Assembly**

• The following parts are required for phase four assembly: #6, #7, #8, #9, #10, #11, #12, #13, #18, #33, #40, #41
• The build of the tail section will be separated into two parts: the inner ribs and tail spine, silicone-rubber tail poured via a three-part mold.
• Remove all of the residual 3-D printed supports and clean out all of the bolt holes from the inner rib parts.

*Ribs and Tail Spine Assembly*

**Fig. 14:**
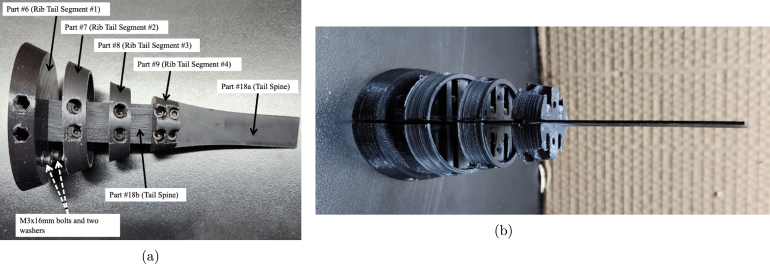
Example images of the final tail ribs and spine assembly. (a) Example image of the final tail rib and spine assembly. The Ribs are oriented such that the largest is closest to the body, and the smallest is placed at the end and connects the two spines together. (b) Top view of how the full tail assembly should look after all the steps are completed.

1. Trace an outline of part #6 (Rib Tail Segment #1) on part #41 Silicone Rubber Sheet and cut out. This will be used as a gasket between the hull and tail segment.
2. Take part #6 (Rib Tail Segment #1) and slide the end of the rectangular section of part #18 (Tail Spine) through the center of the rib. Align the bolt holes and fasten the two parts together using two M3 × 10 mm bolts from part #33. Fig. 14(a) shows the alignment and placement of the ribs in regard the holes on part #18 (Tail Spine).
3. On part #6 (Rib Tail Segment #1), place two M3 × 16 mm bolts and two washers from part #33 through the bottom bolt holes, which will be used to secure the tail to the bottom hull/shell when fully assembled. The bolt placement of the M3 × 16 mm bolts on part #6 (Rib Tail Segment #1) can be seen in Fig. 14(a) Tip: Use a 1/8 in drill bit to slightly enlarge the diameter of the bottom bolt holes to allow for ease of assembly.
4. Take part #7 (Rib Tail Segment #2) and slide it over the second set of bolt holes on part #18 (Tail Spine). Fasten the two parts together using two 3 × 10 mm bolts from part #33.
5. Take part #8 (Rib Tail Segment #3) and slide it over the third set of bolt holes on part #18 (Tail Spine). Fasten the two parts together using two 3 × 10 mm bolts from part #33.
6. Take part #9 (Rib Tail Segment #4) and slide it over the fourth set of bolt holes on part #18 (Tail Spine). Fasten the two parts together using two 3 × 8 mm bolts from part #33.
7. The second piece of part #18 (Tail Spine) is the tail segment, which is connected to the back end slot of part #9 (Rib Tail Segment #4). Fasten the two parts together using two 3 × 8 mm bolts from part #33. Fig. 14(a) shows the alignment and placement of this last rib and how #18b and #18 a (Tail Spine) are oriented with respect to each other.

The Rib and Tail Spine Segment is now complete and shown in Fig. 14.

*Instructions for the 2-part silicone tail mold*

**Fig. 15:**
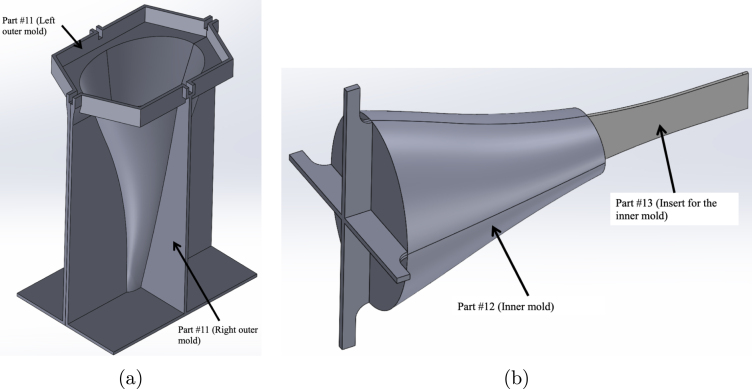
Example images showing the process of steps one and two for the silicone tail mold assembly. (a) Reference image demonstrating how the part #11 (Left/Right of the outer mold) is matched together as described in step one. (b) Reference image showing how part #13 (Insert for the inner mold) fits into part #12 (Inner mold) described in step two.

**Fig. 16:**
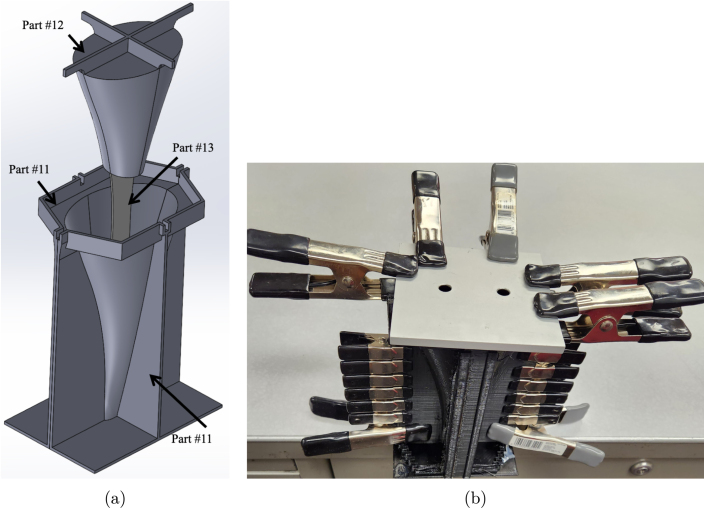
Example and reference images for the tail mold process and what the final set-up before de-molding the tail should look like. (a) Exploded view of the silicone tail mold assembly to show how the combined parts #12 (Inner mold) and #13 (Insert for the inner mold) fit into part #11 (Left/Right of the outer mold). (b) Final tail mold assembly with a weight plate on top of the assembly and numerous clamps to maintain the seal and pressure within the mold.

1. Take part #11 (Left/Right of the outer mold) and use part #40 (Clamp Set) to clamp the two halves together. Ensure that the 3D-print support material is removed from the left/right outer molds to properly place the clamps as close to the interior mold line as possible. Fig. 15 shows how these two faces should meet before clamping.
2. Insert part #13 (Insert for the inner mold) into part #12 (Inner mold). Fig. 15 shows how these two parts fit together and what the final mold insert should look like.
3. In a clean plastic cup, mix equal parts, 28 g of part A and 28 g of part B, of part #10 (Silicone Tail Cover).
4. Once part #10 (Silicone Tail Cover) has been mixed, pour it into part #11 (Left/Right of the outer mold).
5. Insert the combined parts #12 (Inner mold) and #13 (Insert for the inner mold) into part #11 (Left/Right of the outer mold). Ensure the bottom of part #13 (Insert for the inner mold) seats firmly into the open slot at the bottom of part #11 (Left/Right of the outer mold). Place additional clamps around the mold. Fig. 16 shows what the final assembly should look like, including an exploded CAD view and the final tail mold setup with the clamps used. Tip: Use a brick or other weight and place it over the top of the mold to keep the mold insert in place. You may find it helpful to place duct tape on the bottom of the mold to help prevent leaks during curing. Follow the manufacturer’s recommendations for pouring and curing the silicone tail. Once the tail has cured, slowly take the mold apart so as not to tear the material.

**Phase Five: Final Assembly**

**Fig. 17:**
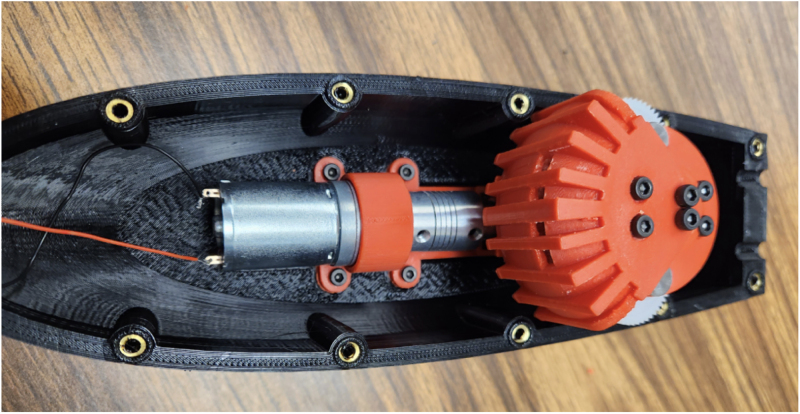
Mounted motor gearbox assembly in the FISHR bottom hull.

**Fig. 18:**
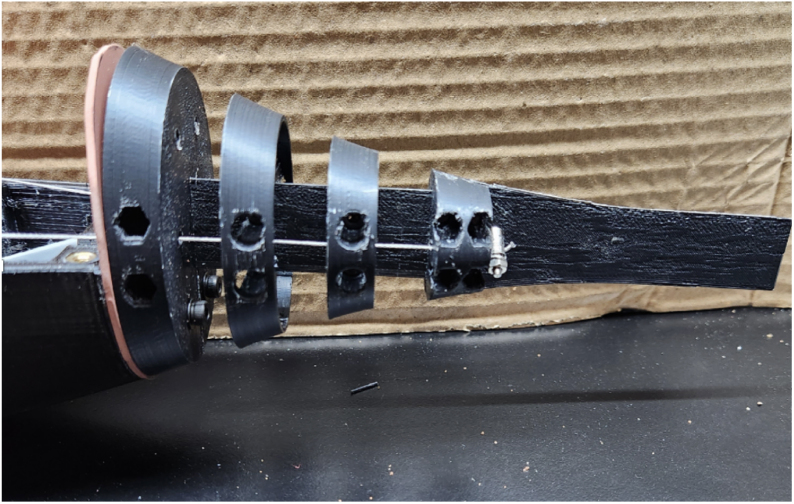
Mounted tail (ribs and spine) assembly mounted to the bottom hull with the traced gasket in between the two parts.

**Fig. 19:**
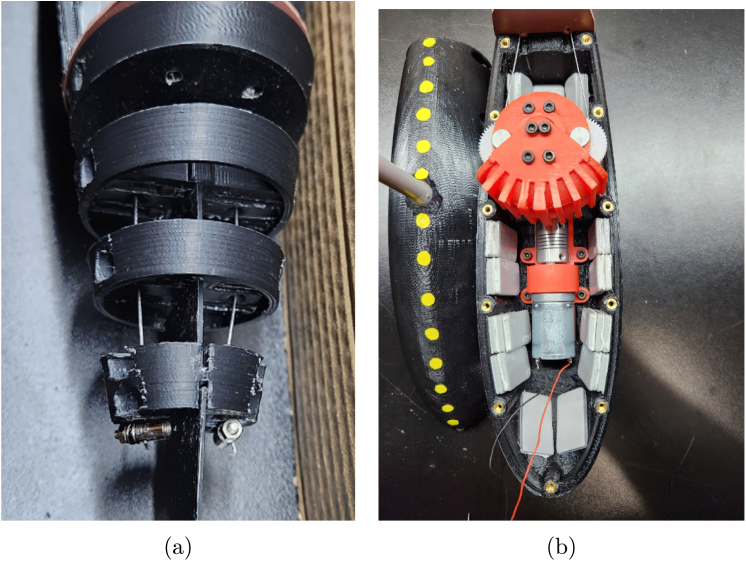
Assembly process for steps five and six, highlighting the wiring of the active tail and weight placement of FISHR. (a) Reference image for step five, which includes the taught wire placed through the tail ribs and the pushrod connectors placed to keep the wire in place. (b) Reference image for step six, which includes the fully assembled bottom hull filled with the weights. (For interpretation of the references to color in this figure legend, the reader is referred to the web version of this article.)

**Fig. 20:**
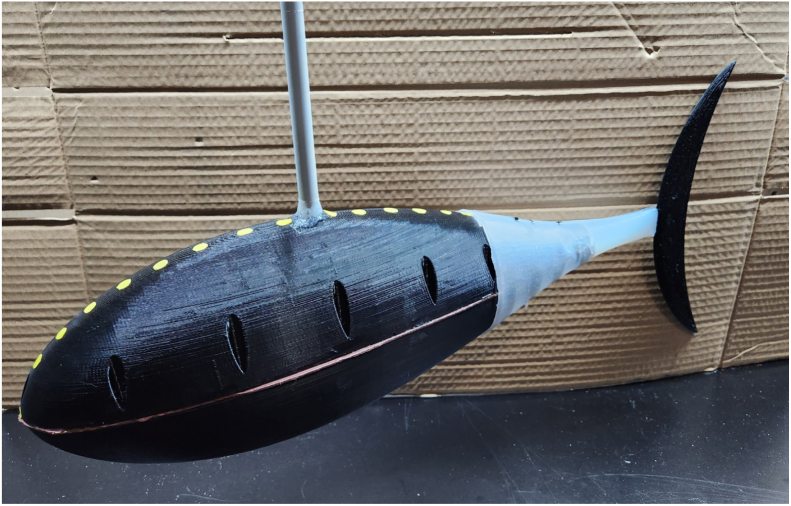
Reference image to show how FISHR should look up to this point in the assembly process after steps seven through ten. Note the gasket material has been placed between the two hulls and has been placed such that the holes align.

• The above four phases will be assembled together, and then additional waterproofing measures will be applied.

1. Take part #37 (12 V Battery Pack) and connect it to part #38 (DC Motor Controller). This will be used to power the motor of the fish.
2. Secure the combined phase one and two, motor and gearbox mount assembly to the four heated inserts on the bottom of part #2 (Bottom Hull) using M3 × 12 mm bolts. Fig. 17 shows the assembly process.
3. Mount Phase Four (Ribs and Tail Spine Assembly) to the M3 threaded heated inserts on the back of part #2 (Bottom Hull) using the two M3 × 16 mm previously installed. Be sure to include the gasket previously cut out between the hull and phase four assembly. Fig. 18 shows the assembly process and includes the position of the tail gasket.
4. Draw the two roughly 6 in braided steel line connected to the pushrod connectors through the center holes of part #6,#7, #8, and #9 (Rib Rail Segments).
5. Using a bolt or another pushrod connector, pull the wire taught and tighten the pushrod connectors or screw to keep the wire in place. Fig. 19(a) shows the final assembled part of this process highlighting the tail wires and pushrod connector. Tip: Connect part the positive and negative leads to part #38 (DC Motor Controller). Turn the motor on and observe the motion of the tail. Often an adjustment of the wires is required to obtain a smooth even motion of the tail. Once the tail moves in a smooth motion, disconnect the positive and negative leads of part # 39 (30-Gauge Red/Black Wire).
6. Use part #36 (Adhesive Wheel Weights) and place 30oz of weights on the side and bottom/top of part #1 and #2 hulls. Fig. 19(b) shows the final assembled bottom hull filled with the weights. Note: More weight will be required to the rear of part #2 (Bottom Hull). Refer to Fig. 19(b). All other weights should be evenly distributed to make the fish neutrally buoyant.
7. Run part #39 (30-Gauge Red/Black Wire) through the top of the hole of part #1 (Top Hull). Note: A solid strut or flexible tubing can be used as a tether for the top of part #1 (Top Hull).
8. Place the gasket cut out from part #14 and place it between part #1 and #2 hulls. Make sure to align the holes from the template to the hulls.
9. Using the M3 bolts from part #33, bolt the two hulls together with the gasket in between.
10. Take the silicone tail formed in phase four and carefully slide it over the rib and tail spine segment that is attached to the hull. Fig. 20 shows what FISHR should look like at this point in the assembly.
11. Use waterproof or electrical tape to tightly secure the silicone tail to the hulls.
12. Place part #3 (Large Caudal Fin) on the tail spine segment. Tip: Apply part # 42 (waterproof super glue) to firmly secure the caudal fin.
13. Apply a layer of part # 42 (waterproof super glue) around the seam of the two hulls.
14. Apply waterproof tape over each individual bolt holes on the top hull.
15. If placing the pectoral, dorsal, and anal fins on the fish, use part # 42 (waterproof super glue) to hold them in place.

**Fig. 21:**
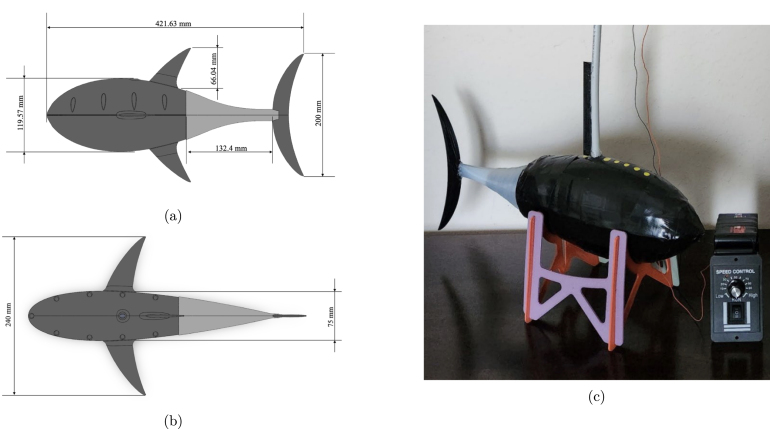
Images of the final FISHR model in CAD with body dimensions (a and b) and the final model of FISHR complete with all the additional taping and waterproofing methods. (a) Side view of FISHR with main body dimensions. (b) Top view of FISH with main body dimensions. (c) Final FISHR model with taping and sealant methods applied.

The assembly is now complete and can be seen in Fig. 21(c), and the constructed model with dimensions is displayed in Fig. 21, Fig. 21.

### 5.1 Construction and 3D printing details

For the fabrication of the robotic fish components, we utilized a range of 3D printers and specific settings to ensure the desired structural integrity and water-tightness. The primary printer used was the Ender 5, configured with factory settings optimized for nylon printing. Cura slicer software was employed, using default nylon parameters to achieve consistent results. Users intending to replicate the build should apply the factory nylon settings in their slicer, adjusting for printer-specific conditions if necessary, such as environmental factors like airflow. The Ender 5 was selected due to its reliable performance in creating large, structurally significant parts.

For smaller internal and tail components, the Bambu Lab X1 Carbon was used, known for its precision in printing finer details. The detailed printing parameters for this printer included a layer height of 2 mm, with a first layer height also set to 2 mm to ensure adhesion. The print settings comprised an aligned seam position, two wall loops, monotonic lines for the top and bottom shell, and a top shell thickness of 0.6 mm. The internal solid infill was set to rectilinear with a 15% sparse infill density and grid pattern. Supports were configured to activate at a 30° threshold angle to accommodate overhangs. For the hull, a 100% infill density was used to ensure maximum robustness.

The 3D printing process involved residual supports to maintain part integrity during printing. Supports were designed to be activated for angles greater than 45°, while flat surfaces, such as the interior of the hull, were adequately supported. Four coats of polyurethane were applied to external components using a foam brush to assist in preventing water intrusion into the hull. Furthermore, to achieve water-tightness, waterproof glue (Gorilla Clear Grip, part # 42) was applied along the seams of the hull and tail sections. Super glue (LOCTITE Ultra Gel Control) was utilized for bonding smaller internal components. For sealing, printable TPU gaskets were experimented with, though their effectiveness was not rigorously quantified. For improved reliability, laser-cut rubber gaskets, derived from the model sketches, are recommended as a more dependable sealing method.

The 3D printing technology and materials utilized in this construction were selected based on their availability and suitability at the time of fabrication. While we employed specific printers and filaments, such as the Ender 5 with nylon and the Bambu Lab X1 Carbon for detailed parts, the choice of materials should not be seen as restrictive. The core principles of construction and waterproofing are consistent across various 3D printing technologies and filaments. It is important to note that achieving water-tightness primarily hinges on the application of appropriate coatings or sealants rather than the specific filament used. Therefore, while the materials and settings described were effective for our build, they are not mandatory for replication. Users are encouraged to adapt the construction process to their available resources, ensuring that adequate sealing measures are implemented to maintain water-tightness regardless of the filament or printer used.

In summary, achieving the necessary water-tightness and structural integrity of the robotic fish involved a combination of precise 3D printing settings, effective use of adhesives, and consideration of potential sealing methods. The detailed parameters provided should assist in replicating and refining the build for similar applications.

## 6 Operation instructions

**Fig. 22:**
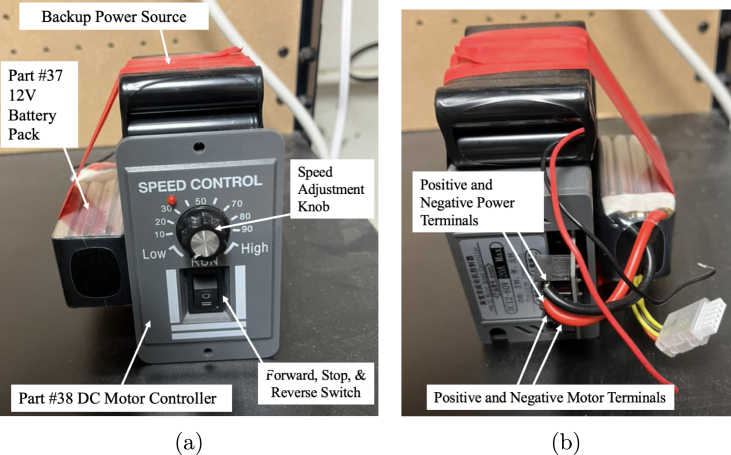
Reference images supporting the setup and operation of Part # 38 DC Motor Controller (DC12 V–60 V/20 A). The device includes a speed control knob for FISHR, and a switch for stop, forward, and reverse functions. (a) Front view of the controller showing Part #37, the 12 V battery pack, and key operational features. (b) Rear view showing terminal connections: two to the power supply (power and ground) and two to the DC motor. (For interpretation of the references to color in this figure legend, the reader is referred to the web version of this article.)

There are many uses and setups for FISHR, so the operating instructions will be dependent on FISHR’s intended use. The basic operating instructions are as follows, given the parts listed in this article. Fig. 22, Fig. 22 support the following setup and instructions.

1. Take part #37 12 V Battery pack, and connect the red (+) and black (–) wire to the respective terminals on part #38 DC Motor Controller (see Fig. 22(b)). This will supply the power to the motor of the fish and allow the user to control the amount of voltage the motor receives, effectively controlling the speed of the robotic fish.
2. Run the red (+) and black (–) wires connected to the motor through the top hull and through a strut or flexible hose, whichever is connected to the top hull.
3. Connect the red (+) and black (–) to the respective terminals on the DC Motor Controller.
4. Turn the DC Motor Controller switch to the I or II position, as shown in Fig. 22(a).
5. Rotate the dial from 0 to High on the DC Motor Controller to the desired speed of the fish.

## 7 Validation and characterization

### 7.1 Waterproofing techniques, design modifications, and validation

The redesign of the FISHR achieved a notable reduction in water intrusion vulnerabilities through comprehensive modifications to the upper and lower hulls. Specifically, the lower hull was reengineered to eliminate all 11 bolt and nut hole openings, resulting in a seamless structure that significantly enhances the fish’s waterproof attributes. Concurrently, the posterior section of the upper hull was modified to integrate a gasket, ensuring a tighter seal with the first tail rib segment and further mitigating potential leaks.

Additionally, we augmented the dimensions of the silicone tail to improve its overlap with the rear of the hulls. This modification reinforced the tail’s integrity and effectively eliminated potential points of water ingress.

To further fortify the waterproofing, we applied a protective coating of polyurethane to critical components. Parts #1 and #2 were each treated with at least four coats of polyurethane and applied using a foam brush for even and consistent coverage. Each coat required approximately 2–4 h to dry before the subsequent layer was applied. This multi-coat process helped to prevent water intrusion into the hull and contributed to the overall water-tight performance of the FISHR.

Our validation process involved testing to assess the impact of these enhancements. Initial trials with the original OpenFish design revealed immediate water seepage, underscoring the need for the modifications. In contrast, the redesigned FISHR underwent successful submerged operation for up to 1 h before any leakage was observed, demonstrating a significant improvement in water-tightness.

#### 7.1.1 Comparison between OpenFish and FISHR

The primary improvements in the FISHR design over the OpenFish include the elimination of bolt and nut openings in the lower hull, the integration of a gasket in the upper hull’s posterior section, and the enhanced silicone tail dimensions. These changes collectively address the previous waterproofing deficiencies and contribute to the FISHR’s enhanced performance in aquatic environments.

The experimental results validate the effectiveness of the FISHR’s redesign in significantly reducing water intrusion and improving overall water-tightness compared to the OpenFish model. The successful extended submerged testing confirms the robustness of the applied changes and substantiates the claims regarding waterproofing enhancements.

### 7.2 Kinematic analysis and overview

It is essential to acknowledge that in thunniform locomotion, it is ideal for there to be little to no head movement in the swimming motion. However, once the FISHR was completed and allowed to swim freely, it was observed that there were head sway motions when operational at higher power percentages. Thus, a kinematic experiment was conducted with the FISHR to compare the ideal tail motion of a thunniform fish to the designed tail motion when head sway was allowed. This kinematic study was done to validate the described swimming locomotion and provide further insight and factors to consider when constructing or improving this model of the robotic fish.

**Fig. 23:**
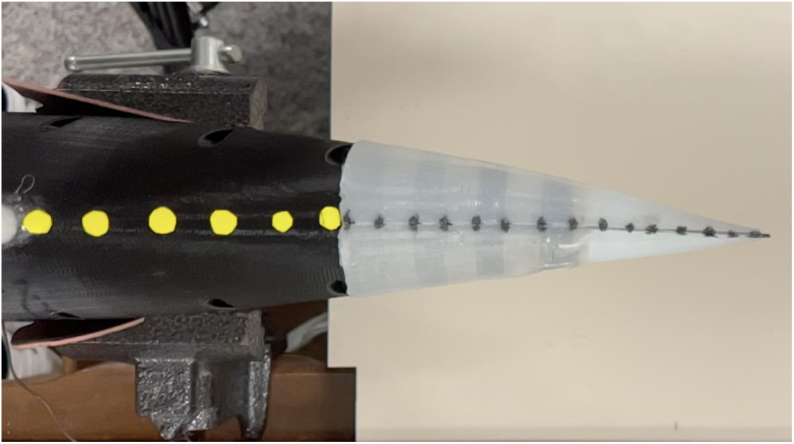
Reference image showing the testing set up for the tail sweep test. FISHR is placed in a vise clamp, allowing only a tail sweep motion. High contrast dots were placed along the centerline of the hull and tail. A high contrast background to the dots was placed below the setup and a camera was set up parallel to FISHR.

First, it was important to validate that the FISHR had a similar or acceptable thunniform locomotion to the theoretical tail sweep motion of a thunniform fish when head sway was limited. For the study with no head sway allowed, Fig. 23 shows the setup where the robotic fish was placed so that the only free-moving section was the active tail of the fish. High contrast dots, as seen in Fig. 23, were added to the midline of the fish from the base to the tip of the tail for motion tracking software. From there, a high-speed camera was set up parallel to the fish and recorded the tail locomotion at a range of speeds, 30% power to 100% power, for three different trials at each speed.

**Fig. 24:**
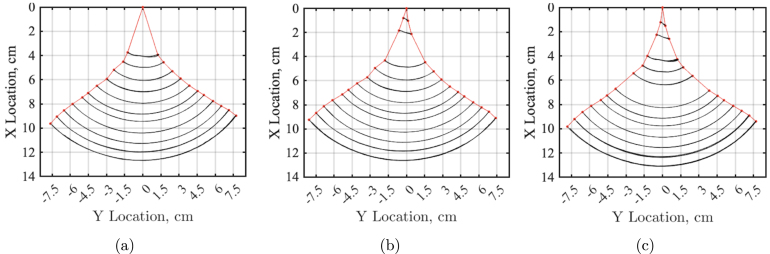
Three trails of FISHR tail sweep motion with no head sway allowed at 100% power. (a) Trial 1 of tail-only motion experiment. (b) Trial 2 tail-only motion experiment. (c) Trial 3 tail-only motion experiment.

The videos were then postprocessed in MATLAB to produce a tail kinematic plot showing the ideal tail sweep area and maximum envelope. Fig. 24 shows the three trials for the tail sweep motion at 100%, which will later be used to compare to the theoretical thunniform locomotion and the kinematic head sway experiments.

This data was then compared to an idealized equation that represents thunniform locomotion, which describes the fish swimming using the product of a sinusoidal function and an amplitude envelope equation for the transverse displacement of the body [16] and is expressed as:

(1) Z(x,t)=A(x)sinkx+2πft

Here, x (cm) is the position coordinate along body length, z(x, t) (cm) is the lateral displacement at time t (s), f (1/s) is the tail-beat frequency, k (1/cm) is the wave number (k =2π/λ), and λ (cm) is the wavelength of a traveling wave. A(x) (cm) is the amplitude envelope and is designed according to the swimming characteristics for thunniform locomotion; as seen in tuna, the starting position of the amplitude envelope A(x) is about 1/3 of the body length L (cm) [16].

(2) $$A\left(x\right)=\left\{\begin{array}{cc} 0 & \text{if}x\leq 1/3L\\ c_{1}\left(x-x_{0}\right)+c_{2}{\left(x-x_{0}\right)}^{2} & \text{if}x>1/3L \end{array}\right)$$

$x_{0}$ (cm) is the starting coordinate of the active, flexible section ($x_{0}=1/3L$), $c_{1}$ (dimensionless) is the linear amplitude envelope, and $c_{2}$ ($m^{-1}$) is the quadratic amplitude envelope. Both $c_{1}$ and $c_{2}$ are adjustable coefficients, typically determined from experimental observations of fish swimming. From historical works, [17], and other studies utilizing the same coefficients, [16], $c_{1}$ is set to 0.00294, and $c_{2}$ is set to −0.1 $\;m^{-1}$. The wavelength k is also set to 1.0L, which resides in the observational data of 0.89–1.29L for most carangiform and thunniform fish [18] and [17]. The flapping frequency was replaced with f = 1/ 4.88 (from observation of FISHR tail motion) to better reflect the FISHR robot.

**Fig. 25:**
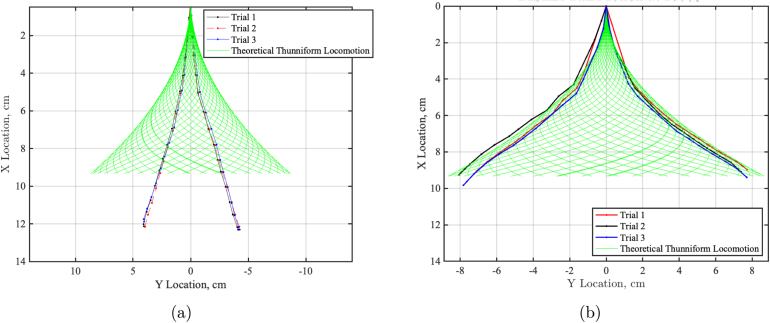
FISHR tail motion at various power settings compared to the theoretical motion envelope. (a) Tail sweep envelopes of FISHR at 30% power (red, blue, and black lines), compared to the idealized tail locomotion for a thunniform fish, green lines. (b) Tail sweep envelopes of FISHR at 100% power (red, blue, and black lines), compared to the idealized tail locomotion for a thunniform fish, green lines. (For interpretation of the references to color in this figure legend, the reader is referred to the web version of this article.)

Fig. 25 shows the tail sweep of FISHR at 100% power compared to the idealized tail locomotion for a thunniform fish using the aforementioned constants and Eqs. (1) and (2).

The figure illustrates that the overall envelope shape of the designed locomotion agrees with the ideal thunniform tail sweep area. However, it should be noted that due to the simple construction of the tail, the motion of the robotic fishtail is different, given that there are fewer hinges for tail flexibility. The design allows the robotic fish to achieve a similar tail sweep area with a more simplified centerline kinematic body wave. The FISHR tail sweep has a circular motion, pivoting from the base of the tail, where the tail remains relatively straight as it oscillates. Meanwhile, a real fishtail will have more flexibility and a curl near the tip of the fin due to the body wave. Additionally, it should be noted that for this kinematic study, when the fish was in lower power modes, the maximum displacement of the tip of the tail was less than the fishtail displacement at 100% power, meaning the fish has a greater swept area at higher powers which was to be expected.

While this study did not directly compare the kinematics of the redesigned robot to its original configuration, it is important to note that the structural components of the tail mechanism remained unchanged in the redesign. The primary focus was to validate that the robot could accurately mimic thunniform locomotion as described in the literature. In particular, our results indicate that the envelope of the tail’s sweep area closely aligns with expected values for thunniform swimmers, underscoring that the redesigned robot maintains a similar movement profile. However, we observed that, instead of exhibiting the more gradual curvature characteristic of thunniform propulsion, the tail motion in the redesigned model relies on a somewhat more hinged movement pattern. The comparison graph of FISHR at 30% power demonstrates the kinematic disparity between this design and theoretical thunniform locomotions. The FISHR design relies more on a flapping hinged motion. This distinction between ideal swimming motion and FISHR’s flapping motion emphasizes that while the general propulsion mechanism is conserved, the motion profile may deviate subtly from ideal thunniform curvature due to mechanical constraints inherent in the tail’s hinge-based design.

**Fig. 26:**
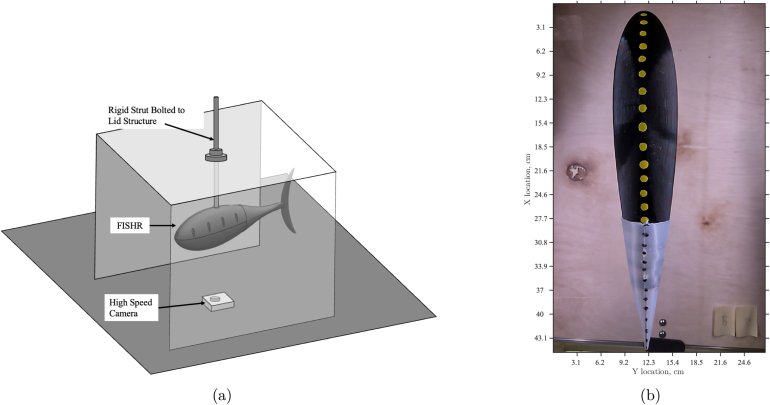
Experimental setup reference image for headway and tail motion sweep test. (a) Graphic depicting the headway and tail sweep motion testing setup. The animation features FISHR secured to the structure, with the high-speed camera positioned below. (b) Experimental setup reference image of FISHR’s full body from a ventral view.

As FISHR is developed to aid in hydrodynamic studies, it is crucial to understand the whole body kinematics when propelling itself forward. Given the simplified kinematics of FISHR and its ability to achieve the envelope of the thunniform locomotion, a tail sweep and head sway kinematic study was conducted to observe how the head sway further impacts the kinematics of FISHR. This setup is shown in Fig. 26, Fig. 26. For this study, FISHR was suspended and not allowed to propel itself forward. The robotic fish was placed on a rigid pole 17 cm from the fastener, holding the fish in place. As before, high-contrast dots were placed along the center line of FISHR to aid motion tracking, and the camera was set up parallel to the fish’s body on the underside to avoid the strut in the imaging process. From there, the camera recorded the locomotion at a range of speeds, 30% power to 100% power, for three different trials at each speed.

**Fig. 27:**
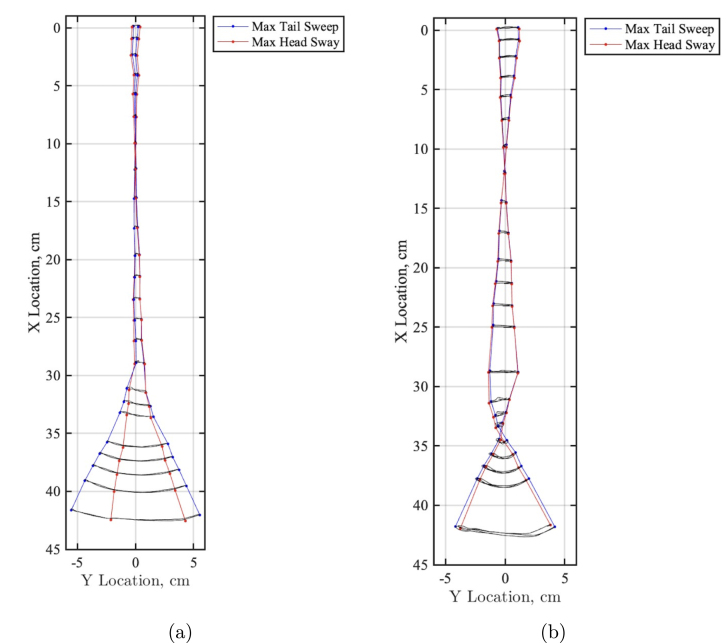
FISHR full-body kinematic motion with head sway allowed at 30% and 100% power. (a) FISHR kinematics with headway shown at 30% power (b) FISHR kinematics with headway shown at 100% power.

Fig. 27 shows the head sway motion at 30% power and 100% power. These plots demonstrate that at 100% power, there is significantly more head sway and a further reduced maximum tail sweep amplitude of 4.17 cm, whereas, at 30% power, there was very minimal head sway and maximum tail sweep amplitude of 5.53 cm. Additionally, the tail sweep amplitude is affected when head sway is allowed. This can be seen in Fig. 24, Fig. 27, where the maximum tail sweep amplitude at 100% is reduced from 7.72 cm to 5.53 cm when head sway is allowed.

**Fig. 28:**
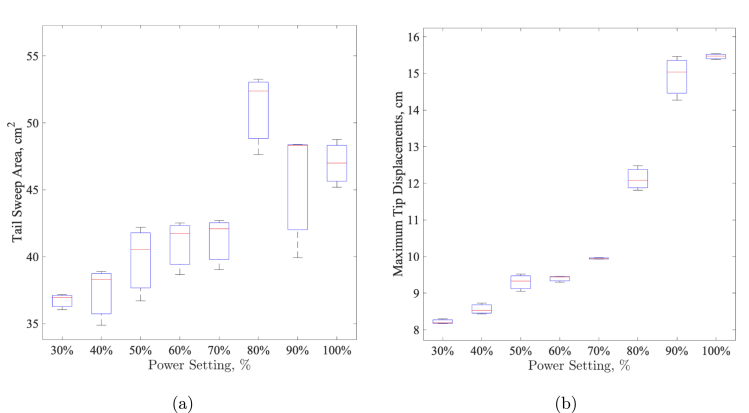
Boxplot results for tail kinematic data: sweep areas and maximum tip displacements across different power settings. (a) Boxplot for Sweep Areas: The plot displays the mean tail sweep area for each power setting (30% to 100%). (b) Boxplot Maximum Tip Displacements: The plot shows the mean maximum tip displacement for each power setting (30% to 100%).

#### 7.2.1 Statistical analysis

To investigate the impact of power settings on movement characteristics, we analyzed differences in both sweep area and maximum tip displacement across several experimental groups. A one-way ANOVA confirmed statistically significant differences across groups for both variables (p = 0.0001 for sweep area andp = 0.0000 for maximum tip displacement), supporting the hypothesis that power settings influence movement dynamics. The boxplot showing this data is shown in Fig. 28. Pairwise t-tests further identified specific group comparisons with significant differences. For sweep area, the values at 30% power showed significant differences compared to the values at 70%, 80%, 90%, and at full power (all p<0.05), with Cohen’s d values indicating substantial effect sizes, particularly between values at 30% power and at full power (d = −7.71) and values at 30% power and at 80% power (d = −6.58). Maximum tip displacement similarly revealed significant differences between values at 30% power and all other groups, with Cohen’s d reaching up to −96.22 for values at 30% power vs. values at full power, illustrating large practical differences in tip displacement. Bootstrapping of the mean sweep area (42.49 $\;{\mathrm{cm}}^{2}$) and tip displacement (10.99 cm) provided additional robustness to the central tendency measures. Collectively, these results strongly indicate that increased power settings led to greater sweep areas and tip displacements, confirming the substantial impact of power on the shape and extent of movement.

Overall, the head sway cases show that the tail sweep area will be impacted when allowing head sway to occur and must be considered when completing experiments. The head sway cases show the input power had a substantial effect on the tail sweep and the head sway of the fish. For instance, at 30%, the maximum head sway amplitude is 0.70 cm, and at 100%, the maximum head sway amplitude is 1.86 cm. It is important to note that there are two points where the motion crosses over itself. One where the fish is pivoting due to the placement of the strut and another one on the tail due to the combined motion of total body sway and the oscillating tail motion. Additionally, it would be essential to consider the length the fish was suspended from the fastener as that could also impact the head sway.

These studies were done to validate the motion of FISH and used to verify that the tail sweep was even and smooth. For future experiments, more consideration should be given to completing a head sway test in water flow to observe how the additional resistance of water impacts the kinematics of the fish.

### 7.3 Limitations and future work

The experimental results demonstrated the kinematic accuracy of FISHR’s motion envelope; however, several limitations highlight areas for future development. The tail’s current design exhibits a hinged motion at the tail base rather than the desired thunniform curvature, which is constrained by the rigid spine structure in the active wire-driven tail. This limits the robot’s resemblance to biological thunniform swimming. Additionally, FISHR’s depth control remains imprecise, as the current design lacks dedicated active dive-plane mechanisms and a refined control system. Furthermore, FISHR’s autonomy is limited, as it cannot independently make decisions based on environmental data, restricting its potential for dynamic or uncontrolled aquatic environments.

Regarding data collection, FISHR cannot record or store environmental or performance data, which hampers its use for research and analysis. Lastly, the motor control system is limited to basic on/off functionality, providing minimal control over swimming phases or directional movement. Further testing and refinement are required, particularly in relation to flow field analysis around the robot, as FISHR is not yet capable of recording quantifiable environmental data.

In addition to the control and mechanical limitations, the current study lacks a hydrodynamic analysis including flow visualizations, drag coefficients, and propulsion efficiency. These metrics will allow a complete validation of FISHR’s swimming performance. While this paper focuses on establishing kinematic accuracy, future work will incorporate preliminary hydrodynamic measurements, including particle image velocimetry (PIV), thrust-drag balance experiments in a towing tank, and free swimming analysis. These experiments will aim to quantify the flow structures and energy efficiency, thereby strengthening the link between biological swimming strategies and FISHR’s design.

Future iterations of FISHR aim to address current limitations and expand its capabilities, broadening its scope for applications in environmental monitoring and marine biology studies. Several design modifications and enhancements are planned to improve performance. To achieve a more thunniform-like tail motion, the active wire-driven tail will be redesigned by replacing the rigid spine with linked hinges and modified ribs, providing smoother and more biologically accurate movement. Depth control will be enhanced by integrating additional pectoral fin motions, controlled by separate motors to function as dive planes, enabling finer control and greater maneuverability.

Autonomy will be significantly improved by incorporating external pressure sensors that collect environmental data, which will be fed into a control loop to adjust tail and fin motions in real time, allowing for autonomous depth control and smarter decision-making based on the robot’s surroundings. To support research and monitoring, FISHR will be equipped with onboard sensors and storage systems to record and store quantitative environmental data during operations. Lastly, the motor system will be upgraded to include stronger waterproof motors with phase control capabilities, providing finer adjustments to swimming phases and improved overall control. These modifications will make FISHR a more robust, versatile, and intelligent robotic fish, capable of overcoming current limitations and enabling new applications in bioinspired robotics.

### 7.4 Conclusions

In conclusion, this paper presents the Fluid Interaction Study: Hydrodynamic Robot (FISHR), an advanced iteration of the OpenFish platform designed to overcome previous limitations in waterproofing, assembly complexity, and experimental validation. While maintaining the core biomimetic principles and propulsion mechanics of OpenFish, FISHR introduces several significant enhancements. These include a seamless lower hull, an improved gasket-based sealing mechanism, and a reinforced silicone tail. These structural improvements simplify the robot’s construction, reduce vulnerabilities to water intrusion, and enhance its durability during operations.

The kinematic analysis and validation experiments conducted on FISHR highlight its successful adaptation of thunniform locomotion, with the tail sweep area closely aligning with expected values for thunniform swimmers. The study also revealed the impact of power settings and head sway on the robot’s movement, demonstrating the importance of considering these factors for accurate kinematic modeling. Despite slight deviations from ideal thunniform propulsion due to the tail’s hinge-based design, FISHR maintains a comparable movement profile, validating its design as a reliable and efficient model for bioinspired locomotion.

Furthermore, FISHR’s performance as a platform for hydrodynamic research is underscored by its ability to facilitate precise adjustments to key design parameters, such as tail length, rigidity, and fin configurations, making it an ideal tool for studies on oscillating propulsion. These findings contribute to a broader understanding of bioinspired robotics and set a new benchmark for open-source robotic fish designs. FISHR advances the capabilities of underwater exploration and ecological studies and improves the accessibility, reliability, and reproducibility of experimental setups, empowering researchers to further develop aquatic robotic systems. Through these enhancements, FISHR bridges critical gaps in the field and lays the groundwork for future innovations in robotic fish technology.

## CRediT authorship contribution statement

**Montana Ligman:** Writing – review & editing, Writing – original draft, Visualization, Validation, Software, Investigation, Formal analysis. **Kioumars A. Rezaie:** Writing – original draft, Visualization, Methodology, Investigation. **Ramya Shah:** Writing – original draft, Methodology, Investigation. **Chris Keeter:** Methodology. **Bryson Sutterfield:** Writing – original draft. **Mirjam Fürth:** Writing – review & editing, Supervision, Conceptualization.

## Declaration of competing interest

The authors declare that they have no known competing financial interests or personal relationships that could have appeared to influence the work reported in this paper.

## Appendix List of URLs to each non-3D printed part

• Part #10 - 2-Part Silicone Resin: https://www.amazon.com/LETS-RESIN-Silicone-Translucent-Non-Toxic/dp/B07V5FFPWC/ref=asc_df_B07V5FFPWC/?tag=hyprod-20&linkCode=df0&hvadid=366309041989&hvpos=&hvnetw=g&hvrand=2556015410835328304&hvpone=&hvptwo=&hvqmt=&hvdev=c&hvdvcmdl=&hvlocint=&hvlocphy=9027902&hvtargid=pla-818695646577&psc=1&tag=&ref=&adgrpid=79166367074&hvpone=&hvptwo=&hvadid=366309041989&hvpos=&hvnetw=g&hvrand=2556015410835328304&hvqmt=&hvdev=c&hvdvcmdl=&hvlocint=&hvlocphy=9027902&hvtargid=pla-818695646577
• Part #24a - 12 V DC Motor 9.7:1: https://www.pololu.com/product/3202/specs
• Part #24b - 12 V DC Motor with Encoder: https://www.amazon.com/Motor-Encoder-Speed-Gearmotor-Robotics/dp/B07GNDG2NC
• Part #25 - Motorshaft Coupler 4 × 4 mm: https://www.amazon.com/uxcell-Aluminum-Coupling-Flexible-Connector/dp/B07G6QBR8Y?th=1
• Part #26 - M0.5 20T Spur Gear: https://www.mcmaster.com/gears/number-of-teeth~20/plastic-gears-and-gear-racks-20-pressure-angle/module~0-5/
• Part #27 - M0.5 50T Spur Gear: https://www.mcmaster.com/gears/plastic-gears-and-gear-racks-20-pressure-angle/module~0-5/number-of-teeth~50/
• Part #28 - L-Gearbox (Huco 332.31.2 Huco L Gearbox, 1:1 Gear Ratio, 0.68 Nm Maximum Torque): https://us.rs-online.com/product/huco/332-31-2/70748865/
• Part #29 - M5 Washer: jhttps://www.amazon.com/uxcell-Nylon-Washers-Screw-Thick/dp/B07NRXR8GY?th=1
• Part #30 - Assorted M3 Spacers: https://www.amazon.com/Electronics-Salon-Spacer-Assortment-Screws-Plastic/dp/B014KJERV4/ref=sr_1_2?crid=37DGQFVIC9EK&keywords=spacer+bolts+for+rc&qid=1682105596&sprefix=spacer+bolts+for+rc
• Part #31 - D1.3 mm Pushrod Connector: https://www.amazon.com/Hobbypark-Adjustable-Connector-Stoppers-Airplane/dp/B01EE6W2TW/ref=sr_1_1_sspa?hvadid=617096904643&hvdev=c&hvlocphy=9027902&hvnetw=g&hvqmt=e&hvrand=104907880314206814&hvtargid=kwd-337868550415&hydadcr=4120_13263594&keywords=rc+pushrod+connectors&qid=1682101303&sr=8-1-spons&psc=1&spLa=ZW5jcnlwdGVkUXVhbGlmaWVyPUExUUxZWkhaM0RRMTNSJmVuY3J5cHRlZElkPUEwMTE4ODYyM1AwWEtDNUs2NUpXSiZlbmNyeXB0ZWRBZElkPUExMDMxMTQyMjZPN0ZPQzNNMEIyVSZ3aWRnZXROYW1lPXNwX2F0ZiZhY3Rpb249Y2xpY2tSZWRpcmVjdCZkb05vdExvZ0NsaWNrPXRydWU=
• Part #32 - M5x20 mm Binding Screw: https://www.amazon.com/QIRUIRED-Stainless-Internal-Cylindrical-Locking/dp/B0C1G88Z7V/ref=sr_1_21_sspa?crid=2F80RBR2B8PWC&keywords=m5+x+20mm+rivet+screws&qid=1687354972&sprefix=m5+x+20mm+rivet+screws
• Part #33 - M3 Assorted Bolts, Nuts, and Washers: https://www.amazon.com/Kadrick-Aassortment-4mm-40mm-Commonly-Stainless/dp/B0BPFXP2M9/ref=sr_1_1_sspa?crid=3UB2R2IMM33LM&keywords=m3+assortment&qid=1688957755&sprefix=m3+assort
• Part #34 - M3 Threaded Heated Inserts: https://www.amazon.com/Printing-Embedment-Knurled-Automotive-Notebook/dp/B09KZSJS88/ref=sr_1_1_sspa?keywords=m3+threaded+heat+inserts&qid=1688612052&sprefix=M3+threated+heat
• Part #35 - Eagle Claw 12” 30lb Braided Steel Fishing Leader: https://www.walmart.com/ip/Eagle-Claw-12-30-lb-Heavy-Duty-Wire-Leader-Bright-6-Pack/16522743?athbdg=L1600&from=/search
• Part #36 - Adhesive Wheel Wights 1/2 oz: https://www.amazon.com/dp/B07FGWKMGP/ref=sspa_dk_detail_3?pd_rd_i=B0851JRMCT&pd_rd_w=RlxtC&content-id=amzn1.sym.dd2c6db7-6626-466d-bf04-9570e69a7df0&pf_rd_p=dd2c6db7-6626-466d-bf04-9570e69a7df0&pf_rd_r=GF99MFV0F87P3P537SNF&pd_rd_wg=bBgSY&pd_rd_r=29588cdf-e7dd-45d8-8c16-bfafc4c01aa2&s=automotive&sp_csd=d2lkZ2V0TmFtZT1zcF9kZXRhaWxfdGhlbWF0aWM&spLa=ZW5jcnlwdGVkUXVhbGlmaWVyPUFQMTFINEFXVzJORlImZW5jcnlwdGVkSWQ9QTEwMjAwMjgxVzBYTzAyMFBLSUhWJmVuY3J5cHRlZEFkSWQ9QTA5MjY5MTExTFQyWFMzWFpDNEdKJndpZGdldE5hbWU9c3BfZGV0YWlsX3RoZW1hdGljJmFjdGlvbj1jbGlja1JlZGlyZWN0JmRvTm90TG9nQ2xpY2s9dHJ1ZQ&th=1
• Part #37 - 12 V Battery Pack: https://www.amazon.com/Tenergy-Capacity-Rechargeable-Replacement-Equipments/dp/B077Y9HNTF/ref=sr_1_7?keywords=small
• Part #38 - DC Motor Controller: https://www.amazon.com/dp/B07TSJ3XPQ?ref_=cm_sw_r_mwn_dp_WVYDAM6PKZS12GDDCZTC
• Part #39 - 30 Gauge Red/Black Wire: https://www.amazon.com/BNTECHGO-Silicone-Flexible-Strands-Stranded/dp/B01M7ZBW2X/ref=sr_1_1_sspa?crid=ORX0SZXZ8P9C&keywords=30
• Part #40 - Clamp Set: https://www.amazon.com/AmazonBasics-20-Piece-Steel-Spring-Clamp/dp/B07ZTWLZ5J/ref=sr_1_1_ffob_sspa?keywords=clamps&qid=1681157101&sr=8-1-spons&spLa=ZW5jcnlwdGVkUXVhbGlmaWVyPUFCTVIxU1k0VlZNWDEmZW5jcnlwdGVkSWQ9QTAyNjkzMTkxVFpJVlNJQjhDRUE0JmVuY3J5cHRlZEFkSWQ9QTA2ODg5MDMzOE5YUjJMSkxLTEREJndpZGdldE5hbWU9c3BfYXRmJmFjdGlvbj1jbGlja1JlZGlyZWN0JmRvTm90TG9nQ2xpY2s9dHJ1ZQ&th=1
• Part #41 - Silicone Rubber Sheet: https://www.amazon.com/Neoprene-Resistant-Silicone-Material-Protection/dp/B09NX8GX5K/ref=sr_1_7_sspa?crid=2BS8FU3PPJMD5&keywords=thin+waterproof+gasket+material&qid=1688609965&sprefix=thin+waterproof+gasket+material
• Part #42 - Gorilla Clear Grip 3-fl oz Liquid Bonding Waterproof, Quick Dry, Flexible Multipurpose Adhesive: https://www.lowes.com/pd/Gorilla-Gorilla-3-fl-oz-Clear-Grip/1002695092
• Part #43 - LOCTITE Ultra Gel Control 5-g Gel Super Glue: https://www.lowes.com/pd/LOCTITE-Ultra-Gel-Control-5-gram-Super-Glue-Clear-Multipurpose-Adhesive/1000461853
